# Integration of Brain and Skull in Prenatal Mouse Models of Apert and Crouzon Syndromes

**DOI:** 10.3389/fnhum.2017.00369

**Published:** 2017-07-25

**Authors:** Susan M. Motch Perrine, Tim Stecko, Thomas Neuberger, Ethylin W. Jabs, Timothy M. Ryan, Joan T. Richtsmeier

**Affiliations:** ^1^Department of Anthropology, Pennsylvania State University University Park, PA, United States; ^2^Center for Quantitative Imaging, Penn State Institutes for Energy and the Environment, Pennsylvania State University University Park, PA, United States; ^3^High Field MRI Facility, Huck Institutes of the Life Sciences, Pennsylvania State University University Park, PA, United States; ^4^Department of Bioengineering, Pennsylvania State University University Park, PA, United States; ^5^Department of Genetics and Genomic Sciences, Icahn School of Medicine at Mount Sinai New York, NY, United States

**Keywords:** morphological integration, brain, skull, Crouzon syndrome, Apert syndrome, craniosynostosis, development, craniofacial

## Abstract

The brain and skull represent a complex arrangement of integrated anatomical structures composed of various cell and tissue types that maintain structural and functional association throughout development. Morphological integration, a concept developed in vertebrate morphology and evolutionary biology, describes the coordinated variation of functionally and developmentally related traits of organisms. Syndromic craniosynostosis is characterized by distinctive changes in skull morphology and perceptible, though less well studied, changes in brain structure and morphology. Using mouse models for craniosynostosis conditions, our group has precisely defined how unique craniosynostosis causing mutations in *fibroblast growth factor receptors* affect brain and skull morphology and dysgenesis involving coordinated tissue-specific effects of these mutations. Here we examine integration of brain and skull in two mouse models for craniosynostosis: one carrying the FGFR2c C342Y mutation associated with Pfeiffer and Crouzon syndromes and a mouse model carrying the FGFR2 S252W mutation, one of two mutations responsible for two-thirds of Apert syndrome cases. Using linear distances estimated from three-dimensional coordinates of landmarks acquired from dual modality imaging of skull (high resolution micro-computed tomography and magnetic resonance microscopy) of mice at embryonic day 17.5, we confirm variation in brain and skull morphology in *Fgfr2c*^*C342Y*/+^ mice, *Fgfr2*^+/*S252W*^ mice, and their unaffected littermates. Mutation-specific variation in neural and cranial tissue notwithstanding, patterns of integration of brain and skull differed only subtly between mice carrying either the FGFR2c C342Y or the FGFR2 S252W mutation and their unaffected littermates. However, statistically significant and substantial differences in morphological integration of brain and skull were revealed between the two mutant mouse models, each maintained on a different strain. Relative to the effects of disease-associated mutations, our results reveal a stronger influence of the background genome on patterns of brain-skull integration and suggest robust genetic, developmental, and evolutionary relationships between neural and skeletal tissues of the head.

## Introduction

Brain and skull shape track one another closely over evolutionary and developmental time. Evidence for the tight morphological correspondence between brain and skull is also seen in conditions classified as human diseases of the skull (e.g., craniosynostosis) or of the brain (e.g., holoprosencephaly, microcephaly), even when both tissues are affected. These observations support the idea that the development of the brain and of the skull is guided by tissue-specific genetic factors, but that these tissues also respond synchronously to signals (e.g., mechanical forces, mechanically induced cell–cell signaling) generated by growth of neighboring tissues (Richtsmeier and Flaherty, 2013; Lee et al., 2017). Craniosynostosis is a condition of complex etiology affecting approximately 1 in every 2,000–2,500 newborns that typically involves the premature fusion of one or more cranial sutures and includes additional anomalies of the soft and hard tissues of the head (Heuzé et al., 2014; Flaherty et al., 2016). Premature suture fusion can occur as an isolated anomaly, or as part of a complex, though well-defined set of phenotypes that define a syndrome. Whether syndromic or isolated, abnormal forces caused by premature closure of calvarial sutures are thought to contribute to increased intracranial pressure and so treatment is initiated early in development, usually in the first year of life. Treatment is invariably surgical, involving reconstructive procedures of strip craniectomy, cranial vault remodeling, the use of varying appliances, and in some cases endoscopic strip craniectomy with postoperative orthotic molding therapy (Lauritzen et al., 1998; Renier et al., 2000; Chang et al., 2010; Honeycutt, 2014). Because the sole treatment is surgery, even with appropriate early diagnosis, management of these patients is difficult and individualized care often involves repeated surgeries and life-long psychosocial challenges (Mohr et al., 1978; Fearon et al., 2006; Mehta et al., 2010).

Though premature suture closure is a feature in many defined syndromes, we focus on the more commonly identified FGFR-associated craniosynostosis syndromes that include Crouzon and Apert syndromes among others. Activating mutations of fibroblast growth factor receptors (FGFRs) are associated with more than half of craniosynostosis cases with known genetic cause. Although thought to be directly responsible for premature suture closure in these cases, FGFRs are known to contribute broadly to the development of many tissues of the head including cartilage, skin, brain and bone (Ornitz, 2002; Thisse and Thisse, 2005; Yaguchi et al., 2009; Hébert, 2011). Accordingly, other organs and tissues including the brain are affected in craniosynostosis conditions. In addition to the premature closure of cranial sutures, individuals diagnosed with Crouzon and Apert syndromes variably share additional common features including exophthalmos, midface retrusion, cranial base anomalies, and abnormal facies. However, there are notable differences between Crouzon syndrome and Apert syndrome. Most striking is the consistent finding of syndactyly of the hands and feet in Apert, but not Crouzon syndrome. Cognitive effects appear to vary in the two syndromes and despite both disorders being typified by premature closure of the coronal suture (uni- or bi-coronal), infants with Apert syndrome are often macrocephalic and have a midline calvarial defect that includes a patent anterior fontanelle and patent sagittal and metopic sutures for the first 2–4 years of life (Cohen and Kreiborg, 1990).

Cognitive effects in craniosynostosis conditions vary widely- from normal to severe- and surgery can have its own effects on long-term outcome (Blank, 1959; Lefebvre et al., 1959; Kapp-Simon et al., 1993, 2007; Renier et al., 1996; Kapp-Simon, 1998; Yacubian-Fernandes et al., 2004, 2005; Becker et al., 2005; Da Costa et al., 2006; Hashim et al., 2014; Fernandes et al., 2016), but there is currently no clear understanding of the relationship between IQ, genetics and brain malformations in these syndromes (Fernandes et al., 2016). Because the gross morphology of the brain mirrors the shape of the skull in craniosynostosis conditions, it is widely assumed that abnormal brain shape is a response to constraints on skull growth caused by premature suture closure. Additional reported neuroanatomical anomalies in craniosynostosis conditions include increased intracranial volume (Gosain et al., 1995; Anderson et al., 2004; Bristol et al., 2004), megalencephaly (Cohen and Kreiborg, 1990, 1991, 1994; Gosain et al., 1995; Posnick et al., 1995; Cohen and MacLean, 2000), ventriculomegaly (Tokumaru et al., 1996; Pooh et al., 1999; Cohen and MacLean, 2000; Renier et al., 2000; Yacubian-Fernandes et al., 2004; Quintero-Rivera et al., 2006), dysmorphology of the corpus callosum (de Leon et al., 1987; Cohen and Kreiborg, 1990, 1991, 1994; Posnick et al., 1995), anomalies in limbic structure (de Leon et al., 1987; Cohen and Kreiborg, 1990, 1991; Renier et al., 2000; Quintero-Rivera et al., 2006), and in gyral patterning (Cohen and Kreiborg, 1990, 1991). Given the temporal primacy of brain development relative to skull development, it is hard to reconcile many of these subcortical central nervous system anomalies found in craniosynostosis conditions with deformational processes triggered by premature suture closure. Still, the primacy of either brain or skull in head development represents one of biology's “chicken-and-the-egg” causality dilemmas.

In the middle of the last century Olson and Miller (1958) established an analytical approach to the concept of “morphological integration,” providing a protocol for the statistical study of morphological relationships among parts of an organism through the study of covariation or correlation of parts (traits). Integration is traditionally defined as the cohesion among parts resulting from interactions of the biological processes producing the phenotypes under study (Klingenberg, 2008), and is thought to reflect the functional, developmental or mechanistic relationships among parts of an organism. Hallgrimsson et al. (2009) argued that integration exists at the level of developmental mechanism; as a property that structures variation by combining the developmental architecture and variation of any particular sample to produce patterns of correlation or covariation. The realization by these authors that the developmental determinants of integration of complex morphological structures can be many, can overlap in time and in outcome, and can obscure each other's effects (Hallgrimsson et al., 2009) calls for novel analytical approaches to covariation structure that are enriched by what we know about the mechanistic bases for development.

**Figure 1:**
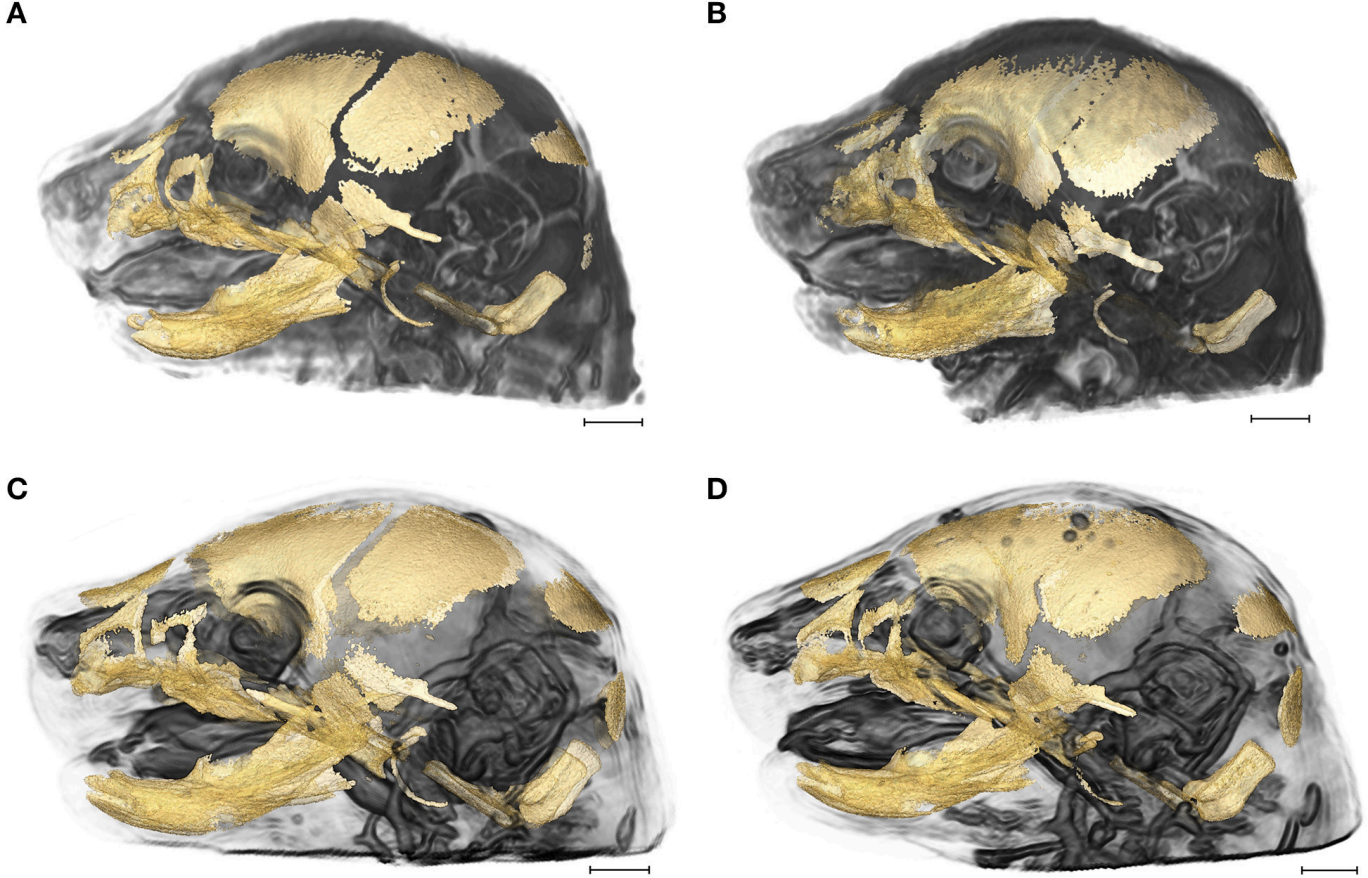
3D reconstructions of high resolution HRμCT images of skull and HRMRM images of brain of E17.5 embryos superimposed to reveal structural associations. **(A)** *Fgfr2*^+/+^ unaffected littermate of the Apert syndrome mouse model; **(B)** *Fgfr2*^+/*S252W*^ Apert syndrome mouse model; **(C)** *Fgfr2c*^+/+^ unaffected littermate of the Crouzon syndrome mouse model; **(D)** *Fgfr2c*^*C342Y*/+^ Crouzon syndrome mouse model. Scale bar = 1 mm. For details of image acquisition see Section Materials and Methods.

The strong correspondence between the global shape of the brain and of the skull across the vertebrates provided the initial incentive to address integration of brain and skull in craniosynostosis conditions (Richtsmeier et al., 2006; Nieman et al., 2012; Richtsmeier and Flaherty, 2013; Rightmire, 2013). There is no doubt that a prematurely closed suture results in postnatal skull deformation that impacts brain shape. But observations of variation in brain shape during late embryogenesis in mouse models for craniosynostosis conditions, with some variation occurring prior to the premature closure of the coronal suture (Aldridge et al., 2010; Martínez-Abadías et al., 2010, 2013a; Motch Perrine et al., 2014), challenge the viewpoint of a skull deformed by premature suture closure as the primary stimulus of brain dysmorphogenesis in craniosynostosis. Here we analyze late prenatal integration of brain and skull in two mouse models of craniosynostosis: the *Fgfr2*^+/*S252W*^ Apert syndrome mouse model (Wang, 2005) and the *Fgfr2c*^*C342Y*/+^ Crouzon syndrome mouse model that carries a mutation common to both Crouzon and Pfeiffer syndromes (Eswarakumar et al., 2004; Figure 1). Both models carry activating FGFR2 mutations and exhibit variation in brain and skull morphology consistent with the corresponding human conditions (Wang, 2005; Perlyn et al., 2006; Martínez-Abadías et al., 2010, 2013a; Motch Perrine et al., 2014). Using analyses of brain and skull integration, we consider the evolutionary, genetic, and developmental basis of variation in brain and skull in model systems for craniosynostosis conditions.

## Materials and methods

### Mouse models

All procedures were reviewed, approved, and carried out in compliance with animal welfare guidelines approved by the Icahn School of Medicine at Mount Sinai and the Pennsylvania State University Animal Care and Use Committees. The generation of the *Fgfr2c*^*C342Y*/+^ Crouzon/Pfeiffer (from here on Crouzon) syndrome mouse and the *Fgfr2*^+/*S252W*^ Apert syndrome mouse models are described elsewhere (Eswarakumar et al., 2004; Wang, 2005). The *Fgfr2*^+/*S252W*^ Apert syndrome mice are maintained on C57BL/6J (B6) inbred background while the *Fgfr2c*^*C342Y*/+^ Crouzon syndrome mice are maintained on a CD1 outbred background. Our sample consisted of 40 E17.5 (embryonic day 17.5) mice: 10 *Fgfr2c*^*C342Y*/+^ Crouzon syndrome mice and 10 of their unaffected littermates; 10 *Fgfr2*^+/*S252W*^ Apert syndrome mice and 10 of their unaffected littermates. E17.5 mice were harvested after euthanasia of the dam with inhalation anesthetics. Specimens were fixed in 4% paraformaldehyde. Genotyping of tail DNA by PCR was performed to distinguish mutant from unaffected littermates.

### Image acquisition and landmark data collection protocols

**Figure 2:**
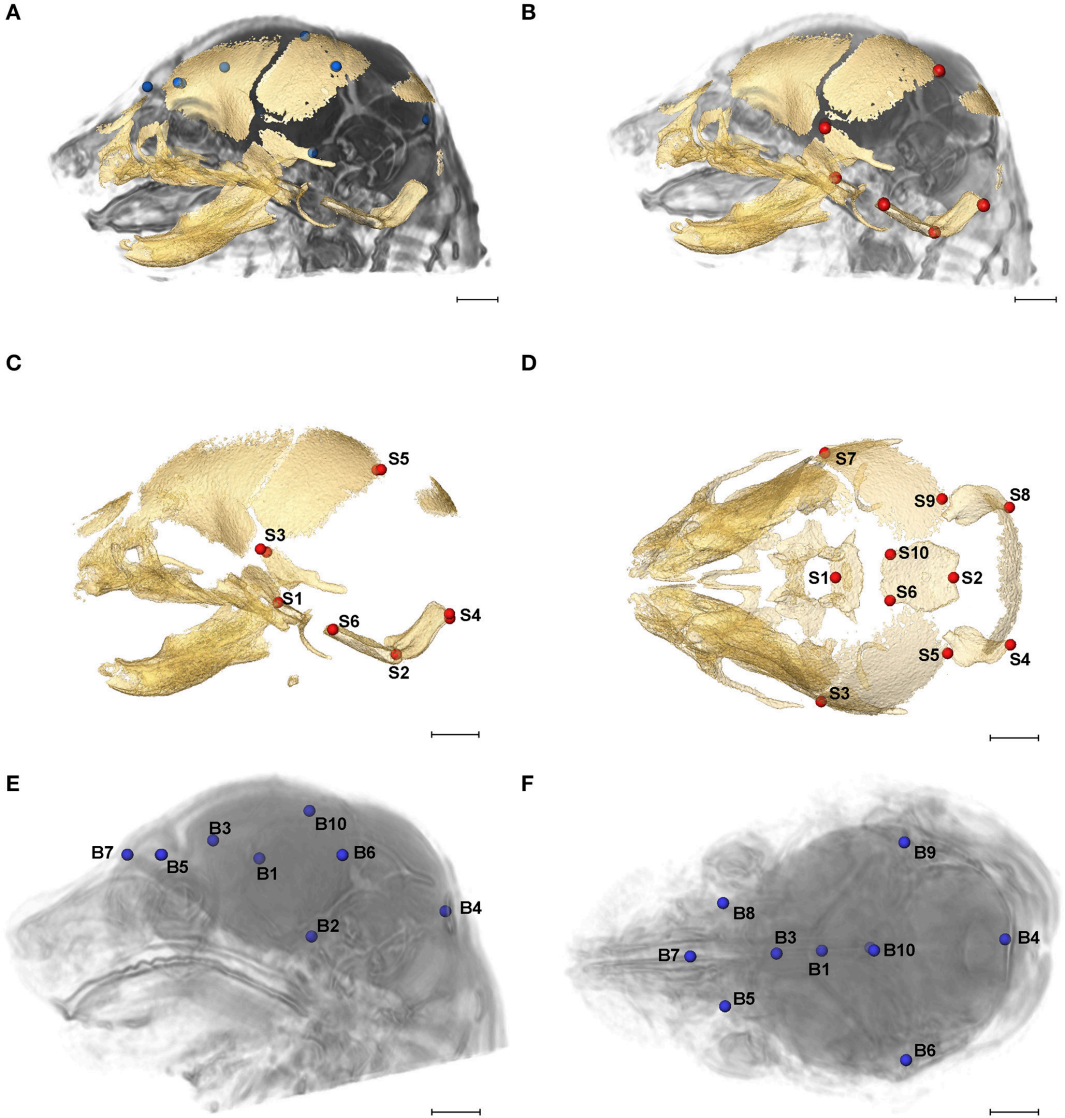
Relative position of brain landmarks (blue dots) and skull landmarks (red dots) used in analysis as positioned on a superimposition of brain and skull (**A, B** lateral view with face to the left). Ten skull landmarks shown on cranial HRμCT isosurface revealing landmarks located on the cranial base (S1, S2, S6, S10) that are visible due to large non-mineralized areas between developing cranial vault bones at E17.5 (**C**, lateral view; **D**, superioinferior view). Ten brain landmarks shown on a HRMRM 3D image reconstruction. Subcortical landmarks (B1, B2, B3) are shown but ghosted (**E**, lateral view; **F**, superioinferior view). Scale bar = 1 mm.

High resolution micro-computed tomography (HRμCT) and magnetic resonance microscopy (HRMRM) images serve as raw data in our analyses (Figure 1). HRμCT images with pixel size and slice thickness ranging from 0.0148 to 0.0168 mm were acquired by the Center for Quantitative Imaging at the Pennsylvania State University using the HD-600 OMNI-X high resolution X-ray computed tomography system (Varian Medical Systems, Inc., Lincolnshire, IL). Image data were reconstructed on a 1,024 × 1,024 pixel grid as a 16 bit tiff but were reduced to 8-bit for image analysis. Isosurfaces were reconstructed to represent all cranial bone at E17.5 based on hydroxyapatite phantoms imaged with the specimens using the software package Avizo 8.1.1 (FEI Company, Inc.). The minimum thresholds used to create the isosurfaces ranged from 70 to 100 mg/cm^3^ partial density hydroxyapatite. A set of 10 three-dimensional (3D) landmarks (Table 1) describing the neurocranium were identified on HRμCT images of each specimen and their 3D coordinate locations (x,y,z) were recorded for use in analysis (Figure 2). Each specimen was digitized twice by the same observer and measurement error was minimized by averaging the coordinates of the two trials. The maximum accepted error in landmark placement was 0.05 mm.

**Table 1:** Landmarks used in analysis of the brain and skull.

**LM No**.	**Abbreviations**	**Skull landmarks used in linear distances**
S1	amsph	Most antero-medial point on the body of the sphenoid
S2	bas	Mid-point on the anterior margin of the foramen magnum, taken on basioccipital
S3	lsqu	Most superior point on the squamous temporal, intersection of the coronal suture, left side
S4	loci	The superior posterior point on the ectocranial surface of the occipital lateralis on the foramen magnum
S5	lpto	Most postero-medial point on the parietal
S6	lsyn	Most antero-lateral point on the corner of the basioccipital
S7	rsqu	Most superior point on the squamous temporal, intersection of the coronal suture, right side
S8	roci	The superior posterior point on the ectocranial surface of the occipital lateralis on the foramen magnum
S9	rpto	Most postero-medial point on the parietal
S10	rsyn	Most antero-lateral point on the corner of the basioccipital
**LM No**.	**Abbreviations**	**Brain landmarks used in linear distances**
B1	ac	Anterior commisure at midline
B2	aptc	Intersection of pons with most caudal aspect of the ventral cerebral surface
B3	gcc	Genu of corpus callosum
B4	midcb	Most caudal point on cerebellar surface
B5	lobc	Most superolateral point of intersection of olfactory bulb with anterior frontal lobe surface, left side
B6	lpol	Most caudolateral point on occipital lobe surface, left side
B7	obnp	Most superiomedial point on the surface of the brain indicating the intersection of the olfactory bulbs with the nasal passages
B8	robc	Most superolateral point of intersection of olfactory bulb with anterior frontal lobe surface, right side
B9	rpol	Most caudolateral point on occipital lobe surface, right side
B10	spcc	Most superiomedial point on the surface of the brain indicating the intersection of the most posterior portions of the left and right halves of the occipital lobes

*Landmark locations are shown in Figure 2*.

HRMRM images were acquired of the same specimens by the High Field MRI Facility at the Pennsylvania State University (Figure 1). The fixed specimens were immersed in 2% Magnevist (Bayer Health Care, Wayne, NJ) phosphor-buffered solution (PBS) for 10 days to reduce the T1 and T2 relaxation times. All experiments were conducted on a vertical 14.1 Tesla Varian (Varian Inc., Palo Alto, CA) imaging system with direct drive technology. To prevent drying and to minimize magnetic susceptibility artifacts during scanning, specimens were immersed in fluorinert liquid, FC-43 (3M, St. Paul, MN). A standard imaging experiment with an isotropic resolution of 80 μm comprised a field of view of 15.4 × 14 × 11 mm^3^ and a matrix size of 192 × 132 (75% partial Fourier: 176) × 137. With eight averages and a repetition time of 75 ms (echo time 25 ms) the total scan time was 3 h. Matlab (The MathWorks, Inc., Natick, MA) was used for image post-processing. By zero-filling all directions by a factor of two, the pixel resolution of a standard imaging experiment was 40 μm^3^. Three-dimensional coordinates of 10 brain landmarks representing surface and subcortical features were identified on each specimen and their coordinate locations (x,y,z) were located on image slices using Avizo 8.1.1 and recorded for use in analysis (Figure 2, Table 1).

### Methods of analysis

#### Principal components analysis of form and shape

Variation in skull and brain morphology in these mouse models during late prenatal stages and at birth have been previously studied by our group (Aldridge et al., 2010; Martínez-Abadías et al., 2013a,b; Motch Perrine et al., 2014). For this study, variation in skull and brain shape for these samples was assessed using principal components analysis (PCA), a data exploration technique that summarizes the variation of a large number of variables in a lower-dimensional space (in our case 90 linear distances from each individual: 45 unique inter-landmark distances estimated from 10 neural landmarks and 45 unique linear distances estimated from 10 skull landmarks). An orthogonal transformation converts the original data to a set of linearly uncorrelated variables that are the principal components. The transformation is ordered such that the first principal component accounts for the largest amount of variance in the data, the second principal component accounts for the second largest amount given the constraint of orthogonality, and so on such that the low-dimensional space is defined by principal component axes that are mutually-orthogonal, linear combinations of the linear distance data. Each observation (individual mouse) is scored for each principal axis and the scores of an observation along the principal axes map that observation into the morphospace defined by the principal component axes.

Two types of PCA were carried out separately for brain and for skull: a PCA based on variation in form (size and shape together), followed by a PCA based on shape variation alone (Darroch and Mosimann, 1985; Jungers et al., 1988; Falsetti et al., 1993). For form, all of the inter-landmark distances for a tissue (brain or skull) were *ln*-transformed and their variance-covariance matrix was used as the basis for the PCA. For shape alone, the linear measures were used to define dimensionless shape variables, where all information about the absolute size of the measurements was removed and only information about proportions remained. The shape variables for an observation were defined as the *ln*-transformed ratios of its linear distances to the geometric mean of all of its distances (where the geometric mean serves as a measure of overall size of either brain or skull). As with the PCA for form, the PCA for shape was based on the variance-covariance matrix. When using these definitions, the amount of overall variance in form can be partitioned into the proportion that is due to form (size and shape) variation and the proportion that is due to variation in shape alone (Darroch and Mosimann, 1985). The amount of variation due to form (size and shape) is the sum of variances for all of the *ln*-transformed linear distances, while the amount of variance due to shape alone is the sum of variances for the *ln*-transformed ratios. The difference in these is the amount of variance due to size alone. All principal components analyses were performed using SAS 9.3 (SAS Institute, Cary, NC). PCA is a data exploration (clustering) technique. More detailed statistical comparisons of brain and skull morphology in craniosynostosis mouse models can be found in previous publications from our group and others (Perlyn et al., 2006; Aldridge et al., 2010; Martínez-Abadías et al., 2010, 2013a; Snyder-Warwick et al., 2010; Motch Perrine et al., 2014).

#### Statistical comparison of morphological integration patterns

Modern quantitative approaches to the study of integration commonly use matrix correlations and/or covariances to explain how biological structures are organized. Typically, *a priori* biological hypotheses about how biological structures are organized are modeled by correlation/covariance matrices and compared with empirical patterns of covariation among traits estimated from the samples under study. Permutation tests are used to evaluate a null hypothesis that the association between two matrices (one *a priori* and one observed) is not different than what would be expected by random chance. Though there are many ways to propose hypotheses of cranial integration based on the analysis of data (see Roseman et al., 2011 for examples), it is common for studies of cranial integration to favor an *a priori* hypothesis that the skull is composed of three modules: cranial vault, cranial base and facial skeleton. A common approach to morphological integration is based on the framework of geometric morphometrics, which is based on analysis of shape variation using the Procrustes superimposition (Gower, 1975; Rohlf and Slice, 1990; Bookstein, 1991; Dryden and Mardia, 1998) under the assumption that size has been “removed” by the superimposition step. However, the alignment of forms accomplished by any superimposition approach is based on the estimation of the nuisance parameters of rotation, translation, and scaling. So, superimposition does not remove differences due to these parameters but instead incorporates them into the analysis and permanently sets these parameters according to the preferred superimposition scheme (Lele and McCulloch, 2002; Richtsmeier et al., 2002). To avoid the use of superimposition to estimate correlation/covariance among traits and differences in these patterns we use linear distances estimated from 3D coordinate locations of biological landmarks (Richtsmeier et al., 2006). By using linear distances, we also circumvent the affine registration (a mapping that includes three translations, three rotations, three scales, and three shears) required to register data from HRMRM brain images to those from HRμCT skull images (Nieman et al., 2012).

Here we explore *differences* in brain-skull integration by between-groups statistical comparison of patterns of correlation/covariance in brain and cranial metrics using a previously published method (Cole and Lele, 2002; Richtsmeier et al., 2006). Our analysis provides information about how typical integration of brain and skull is altered in the presence of craniosynostosis-causing mutations by comparing integration patterns of embryos carrying mutations with those of their unaffected littermates. We also analyze difference in brain-skull integration between the two *Fgfr2* mutants and between the unaffected littermates of both craniosynostosis models.

To statistically compare patterns of brain-skull morphological integration between groups of mice we used a boot-strap based method (Cole and Lele, 2002; Richtsmeier et al., 2006) implemented in MIBoot, a Windows-based software package (Cole, 2002). 3D coordinates of 10 skull landmarks and 10 brain landmarks recorded from HRμCT and HRMRM, respectively, were used to estimate a total of 90 linear distances (45 unique linear distances between brain landmarks and 45 unique linear distances between skull landmarks) that were used in analysis. For each sample, a correlation/covariance matrix was estimated for unique linear distances pairs consisting of one brain and one skull metric (see Supplementary Table 1), and a correlation-difference matrix was estimated by subtracting the elements of the correlation matrix estimated for one sample from the corresponding elements of the matrix estimated for the other sample used in the comparison. Elements of the correlation-difference matrix were statistically evaluated using a non-parametric bootstrap approach. If the correlation matrices are the same for two samples, then the correlation-difference matrix consists of zeros. If they are not similar, the bootstrap is used to estimate confidence intervals for each correlation difference (α ≤ 0.10) (Richtsmeier et al., 2006). If a confidence interval does not include zero (the expected value under the null hypothesis of similarity), then the null hypothesis of equal associations for that particular linear distance pair is rejected. Using this method, we statistically compared the correlation patterns of skull-brain integration between each mutant model and their unaffected littermates, between the *Fgfr2c*^*C342Y*/+^ Crouzon syndrome mice and the *Fgfr2*^+/*S252W*^ Apert syndrome mice, and between the unaffected littermates of the two groups (*Fgfr2c*^+/+^ and *Fgfr2*^+/+^ mice).

## Results

### Skull morphology

#### Morphological differences of *Fgfr2c*^*C342Y*/+^ and unaffected littermates at E17.5

**Figure 3:**
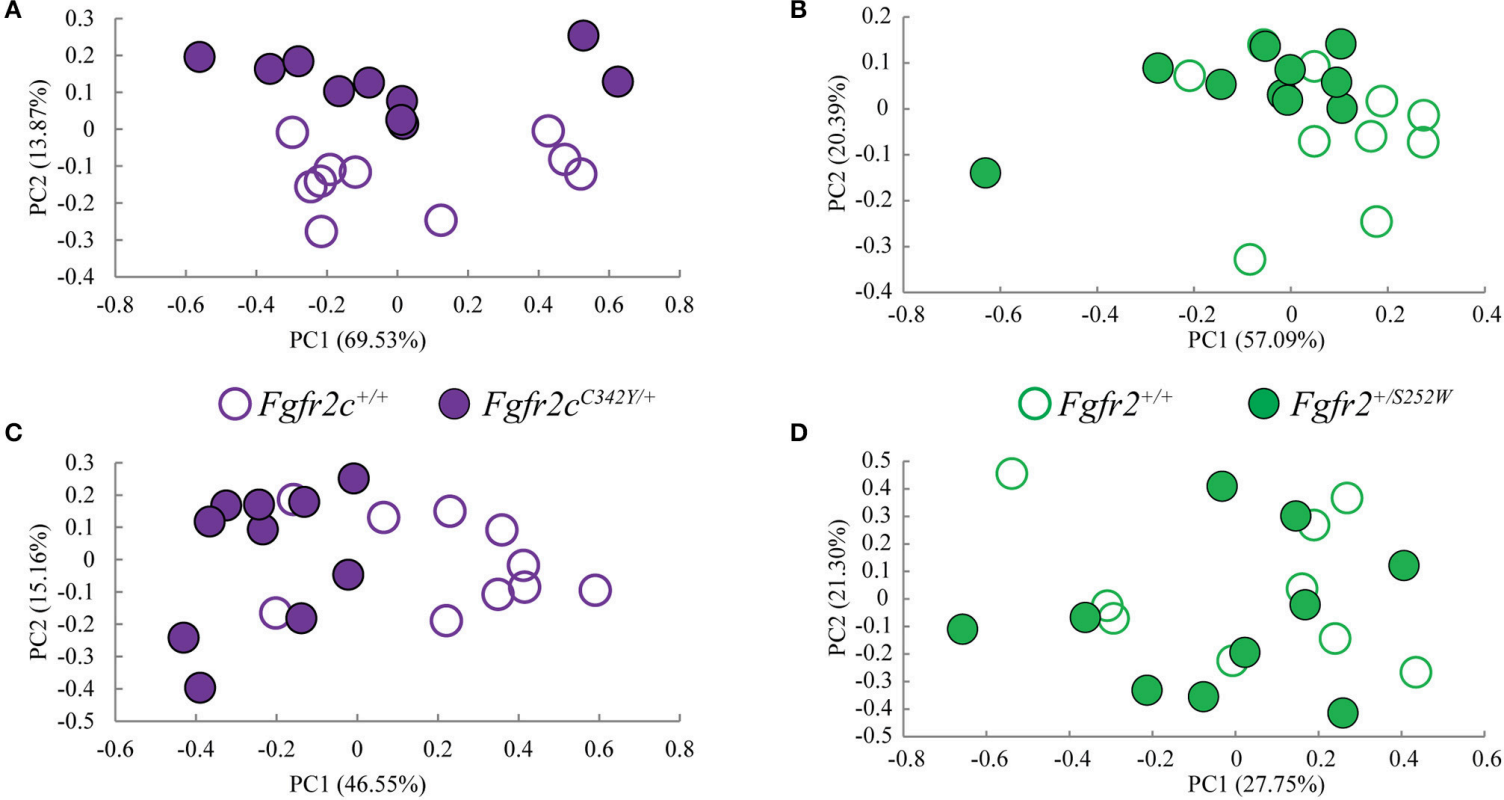
Results of PCA analyses of form based linear distances estimated among landmarks for skull and brain. **(A,B)** Scatter plots of individual scores based on PCA of skull form (shape + size). **(A)** Distribution of *Fgfr2c*^*C342Y*/+^ mutant mice and unaffected littermates (*Fgfr2c*^+/+^) along first and second Principal Components axes (PC1 and PC2) estimated using all unique linear distances among 10 cranial landmarks of each observation, scaled by the observation's geometric mean. **(B)** Distribution of *Fgfr2*^+/*S252W*^ Apert syndrome mice and unaffected littermates (*Fgfr*^+/+^) along first and second Principal Components axes (PC1 and PC2) estimated using all unique linear distances among 10 cranial landmarks of each observation, scaled by the observation's geometric mean. **(C,D)** Scatter plots of individual scores based on PCA of brain form (shape + size). **(C)** Distribution of *Fgfr2c*^*C342Y*/+^ mutant mice and unaffected littermates (*Fgfr2c*^+/+^) along first and second Principal Components axes (PC1 and PC2) estimated using all unique linear distances among 10 brain landmarks of each observation, scaled by the observation's geometric mean. **(D)** Distribution of *Fgfr2*^+/*S252W*^ Apert syndrome mice and unaffected littermates (*Fgfr*^+/+^) along first and second Principal Components axes (PC1 and PC2) estimated using all unique linear distances among 10 brain landmarks of each observation, scaled by the observation's geometric mean.

We considered both the relative amount of variation due to skull form (size and shape) and the relative amount of variation attributable to skull shape alone (i.e., without variation due to skull size) by conducting a PCA based on 45 unique inter-landmark distances estimated from 10 neurocranial landmarks (Figures 2B–D; Darroch and Mosimann, 1985; Jungers et al., 1988; Falsetti et al., 1993). When form is considered, the first PC axis summarizes 69% of variation in skull morphology among of *Fgfr2c*^*C342Y*/+^ Crouzon syndrome mice and unaffected littermates, while PC2 accounts for ~14% of the variation (Figure 3A). When skull shape is considered (i.e., without variation due to skull size) almost all variation is accounted for by PC1 (Supplementary Figure 1A), an indication of the substantial influence of shape in determining the morphological differences among groups. The degree of separation among *Fgfr2c*^*C342Y*/+^ Crouzon syndrome mice and unaffected littermates was similar whether we considered form or shape (although they align along different PCs). Additional analyses of *Fgfr2c*^*C342Y*/+^ Crouzon syndrome mice and unaffected littermates using a full complement of skull landmarks (data not shown) and a bootstrap test for statistical significance demonstrates statistically significant differences in skull morphology between *Fgfr2c*^*C342Y*/+^ Crouzon syndrome mice and unaffected littermates at E17.5. These differences were also present at P0 (Martínez-Abadías et al., 2013a).

#### Morphological differences of *Fgfr2^+/*S252W*^* and unaffected littermates at E17.5

Using linear distances estimated from 10 neurocranial landmarks with each observation scaled by the observation's geometric mean, PCA of the *Fgfr2*^+/*S252W*^ Apert syndrome mice and their unaffected littermates reveal differences between groups on the basis of skull morphology, whether we analyze form (Figure 3B) or shape (Supplementary Figure 1B). Fifty-seven percent of variation is summarized by PC1 when form is considered (Figure 3B), but this increases to 99% when only shape is considered (Supplementary Figure 1). A previously published study using a full complement of landmarks covering the entire skull and larger samples reveal statistically different skull shapes between *Fgfr2*^+/*S252W*^ Apert syndrome mouse models and their unaffected littermates at E17.5 where a null hypothesis of similarity in shape was soundly rejected.

These results confirm that even when small samples and a limited number of landmarks are used in analysis, skull shape is different in each craniosynostosis mouse model relative to unaffected littermates as early at E17.5.

### Brain morphology

#### Morphological differences of *Fgfr2c^*C342Y*/+^* and unaffected littermates at E17

Previous studies from our group suggest that the distinct cranial morphologies of syndromic craniosynostosis conditions result from the effect of specific FGFR mutations on skull development and on additional non-osseous tissues (Martínez-Abadías et al., 2013a,b; Motch Perrine et al., 2014). To consider the relative amount of variation attributable to brain form (size and shape) and brain shape (without variation due to brain size), we conducted a PCA based on all 45 unique inter-landmark distances estimated from 10 brain landmarks (Figures 2A,E,F). Approximately 46% of the variance among individual brains is accounted for by PC1 when form is analyzed (Figure 3C), and this increases to 98% of the variance being accounted for by PC1 when shape is considered (Supplementary Figure 1C). A plot of the first two principal axes of the PCA reveals that brain shape of *Fgfr2c*^*C342Y*/+^ Crouzon syndrome mice and unaffected littermates separate along PC1 (Figure 3C), though the brains of two unaffected littermates cluster with the mice carrying the *Fgfr2* mutation. The separation between groups is similar when shape is considered (Supplementary Figure 1C). This agrees with a statistical analyses of brain morphology in *Fgfr2c*^*C342Y*/+^ Crouzon syndrome mice (using a larger sample and one additional brain landmark) in which we rejected the null hypothesis of similarity in brain shape between *Fgfr2c*^*C342Y*/+^ Crouzon syndrome mice and unaffected littermates (data not shown).

#### Morphological differences of *Fgfr*2*^+/*S252W*^* and unaffected littermates at E17.5

PCA did not reveal a difference in the brains of *Fgfr2*^+/*S252W*^ mice relative to their unaffected littermates at E17.5 whether brain form (Figure 3D) or brain shape (Supplementary Figure 1D) was analyzed. This finding was confirmed by statistical analysis of these data using Euclidean Distance Matrix Analysis (EDMA) (Lele and Richtsmeier, 2001). A previous analysis of brain shape revealed that brains of *Fgfr2*^+/*S252W*^ mice are statistically different from their unaffected littermates at P0 (Aldridge et al., 2010), suggesting that differences in brain morphology of *Fgfr2*^+/*S252W*^ mice are generated between E17.5 and P0.

### Brain-skull integration in two mouse models for craniosynostosis conditions

#### Correlation among brain measures, among skull measures, and between brain and skull is strong across all models

Using the correlation matrices estimated for each sample we considered correlations among all skull linear distances and among all brain linear distances (Supplementary Table 1). The mean and standard deviation of the absolute value of the correlation coefficients for each sample reveal that raw correlations among skull measures, among brain measures, and among brain and skull measures (Table 2) are strong, with comparable standard deviations. These summary statistics suggest that the magnitude of correlations within these cranial tissues is comparable across all samples considered at E17.5. We focus on the brain-skull correlation matrices in our comparative analysis of integration patterns.

**Table 2:** Mean (X) and standard deviation (S) of the raw estimates (white columns) and the absolute value (blue columns) of correlation coefficients for all brain measures, all skull measures, and between all brain and all skull measures for all samples used in analysis.

**Sample**	**Brain linear distances**				**Skull linear distances**				**Brain and skull linear distances**			
			**Absolute value**				**Absolute value**				**Absolute value**	
	***X***	***S***	***X***	***S***	***X***	***S***	***X***	***S***	***X***	***S***	***X***	***S***
*Fgfr2c^+/+^*	0.155	0.831	0.818	0.213	0.138	0.843	0.825	0.219	0.114	0.831	0.811	0.216
*Fgfr2c^*C342Y*/+^*	0.158	0.833	0.809	0.253	0.156	0.820	0.790	0.269	0.118	0.823	0.787	0.266
*Fgfr2^+/+^*	0.109	0.825	0.797	0.238	0.053	0.797	0.753	0.266	0.026	0.805	0.765	0.252
*Fgfr2^+/*S252W*^*	0.130	0.781	0.744	0.271	0.068	0.851	0.820	0.235	0.018	0.806	0.761	0.266

#### Integration of brain and skull is similar in *Fgfr2c*^*C342Y*/+^ crouzon syndrome mice and unaffected littermates

**Figure 4:**
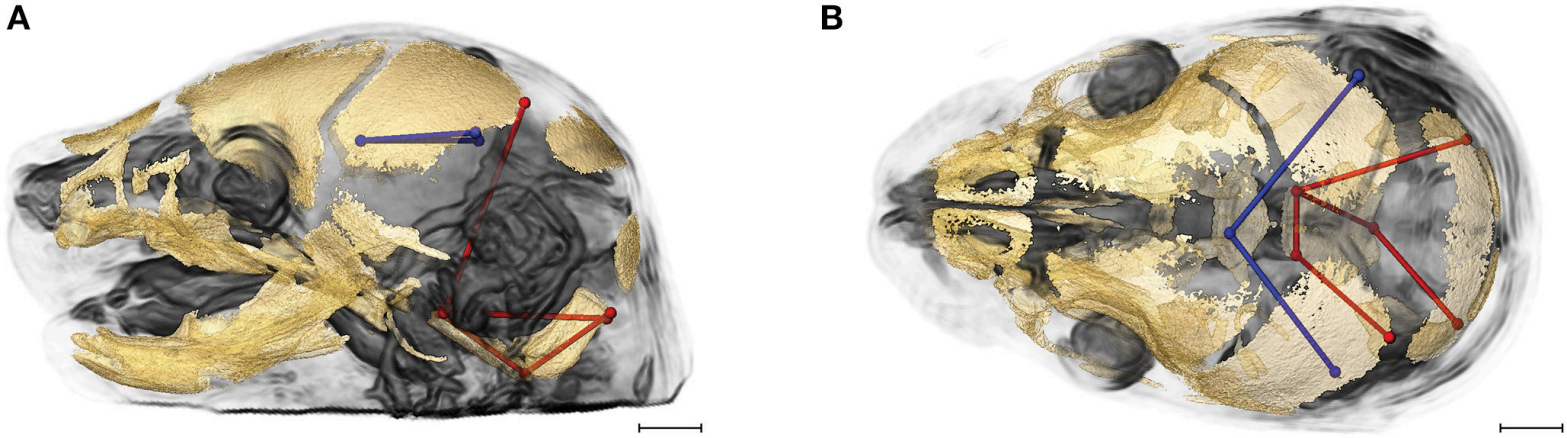
Brain (in blue) and skull (in red) linear distances whose association was statistically significantly different between *Fgfr2c*^*C342Y*/+^ Crouzon syndrome mice and unaffected littermates at E17.5 pictured on HRMRM and HRμCT reconstruction of a *Fgfr2c*^+/+^ unaffected littermate (**A**, lateral view; **B**, superoinferior view). The two brain metrics (BR7 and BR29; see Supplementary Table 2) were in included in 17 of 61 (~28%) of the correlations that were significantly different between *Fgfr2c*^*C342Y*/+^ Crouzon syndrome mice and unaffected littermates. Scale bar = 1 mm.

Evaluation of the correlations among brain and skull measures in *Fgfr2c*^*C342Y*/+^ Crouzon syndrome mice and unaffected littermates reveals a generalized similarity in the magnitudes of correlation. Of the 2,025 correlation coefficients for unique pairs of brain and skull measures, only 61 (3%) are significantly different between mice carrying this *Fgfr2* mutation and their unaffected littermates. Of these differences, 23 show a positive difference (a given correlation is of a greater magnitude in unaffected littermates compared to mice carrying the *Fgfr2* mutations) while 38 of the differences are indicative of brain-skull correlation coefficients that are of a greater magnitude in mice carrying the mutation. Figure 4 shows two linear distances on the midbrain [BR7 (lpol&ac) and BR29 (rpol&ac) (in blue)] that commonly (in 17% of significantly different correlations) have a different association with measures of the cranial base and of posterior neurocranial height (shown in red) in mutant relative to unaffected mice. No correlations between any brain dimensions and measures of the anterior cranial base (sphenoid) are significantly different between groups. These particular differences notwithstanding, our results suggest overwhelming similarity in the strength and pattern of correlation among measures of skull and brain among Crouzon syndrome mice and unaffected littermates.

#### Integration of brain and skull is similar in *Fgfr2*^+/*S*252*W*^ apert syndrome mice and unaffected littermates

Comparison of integration patterns among cranial measures in *Fgfr2*^+/*S252W*^ Apert syndrome mice and unaffected littermates reveals a strong similarity between groups in the magnitudes of correlation among measures of the skull, measures of the brain, and among measures of the brain and skull (Table 2). While the magnitude of the absolute value of correlations among measures on the brain only or between brain and skull are similar between groups, the magnitudes of the absolute value of correlation coefficients among measures of the skull are relatively increased in mice carrying the FGFR2 S252W mutation. This finding corroborates an earlier study of skull integration in *Fgfr2*^+/*S252W*^ and *Fgfr2*^+/*P*253*R*^ mice where it was suggested that FGFR/FGF signaling may be a covariance generating mechanism in skull development that acts as a global factor modulating the intensity of skull integration (Martínez-Abadías et al., 2011).

**Figure 5:**
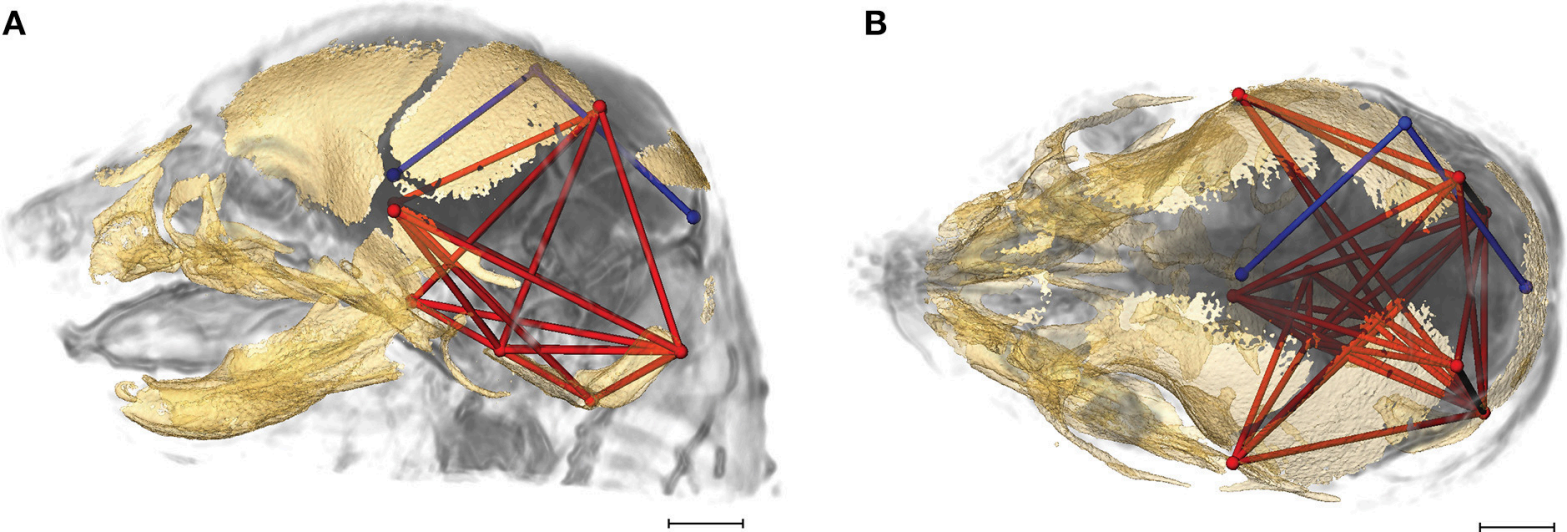
Brain (in blue) and skull (in red) linear distances whose association was statistically significantly different between *Fgfr2*^+/*S252W*^ Apert syndrome mice and *Fgfr2*^+/+^ unaffected littermates at E17.5 pictured on HRMRM and HRμCT reconstruction of a *Fgfr2*^+/+^ unaffected littermate (**A**, lateral view; **B**, superoinferior view). The two brain metrics (BR29 and BR34; see Supplementary Table 2) were in included in 71 of 139 (~51%) of the correlations that were significantly different between *Fgfr2*^+/*S252W*^ Apert syndrome mice and *Fgfr2*^+/*S252W*^ Apert syndrome mice and *Fgfr2*^+/+^ unaffected littermates. Scale bar = 1 mm.

Of the 2,025 correlations among unique pairs of brain and skull linear distances, 139 (6.8%) are significantly different between *Fgfr2*^+/*S*252*W*^ *Apert* syndrome mice and unaffected littermates. Of these differences, 75 show a positive difference (a given correlation is statistically of a greater magnitude in *Fgfr2*^+/*S*252*W*^ Apert syndrome mice relative to unaffected littermates) while 64 of the differences reveal significantly stronger correlations between certain brain and skull linear distances in unaffected littermates. Linear distances most commonly involved in significant differences in integration involve associations of the right hemisphere of the brain [BR29 (rpol&midcb) and BR34 (rpol&ac)] with particular skull measures (Figure 5, blue lines). Though these brain dimensions are included in approximately half (71 of 139) of the linear distance pairs whose correlation differed significantly between *Fgfr2*^+/*S252W*^ Apert syndrome mice and unaffected littermates, dimensions of the left side of the brain are included among other significant results (not pictured). Relative to patterns of significant differences observed between *Fgfr2c*^*C342Y*/+^ Crouzon syndrome mice and unaffected littermates (Figure 4), differences in brain-skull integration patterns in the Apert model include additional measures on the parietal bones, the squamous temporal, and measures of the cranial base rostral to the basi-occipital synchondrosis. These details notwithstanding, overall our results suggest a strong similarity in the strength and pattern of correlation among measures of brain and skull in mice carrying the *Fgfr2* S252W mutation and their unaffected littermates.

#### Comparison of craniosynostosis models reveals increased difference in integration of brain and skull

Given the patterns of overall similarity in covariation of brain and skull between each craniosynostosis syndrome model and their respective unaffected littermates, we wondered how the integration of brain and skull varied between the two mutations groups. Of the 2,025 correlations among unique pairs of skull and brain linear distances, 277 (13.67%) are significantly different between mice carrying one of the two craniosynostosis mutations. Of these differences, 67 show a correlation of greater magnitude in mice carrying the Fgfr2 S252W Apert syndrome mutation while 210 of the differences indicate brain-skull correlations that are of a greater magnitude in mice carrying the Fgfr2c C342Y mutation.

**Figure 6:**
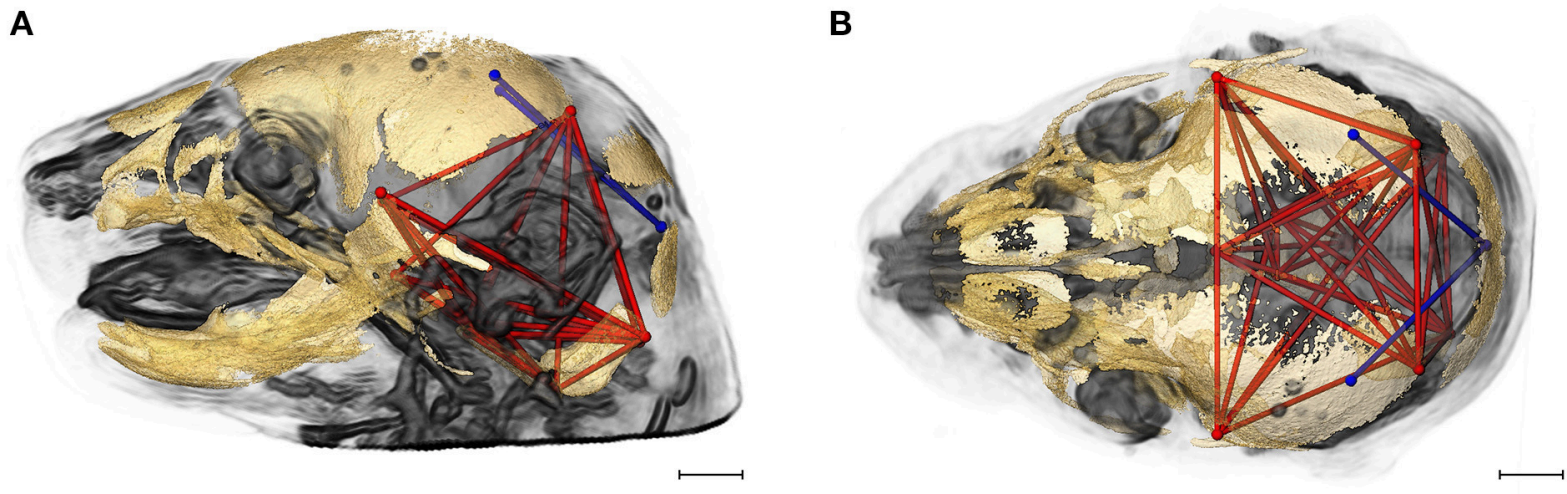
Brain (in blue) and skull (in red) linear distances whose associations were statistically significantly different between *Fgfr2c*^*C342Y*/+^ Crouzon syndrome and *Fgfr2*^+/*S252W*^ Apert syndrome mice at E17.5 pictured on HRMRM and HRμCT reconstruction of a *Fgfr2c*^*C342Y*/+^ Crouzon syndrome mouse (**A**, lateral view; **B**, superoinferior view). Pictured are two brain metrics (blue lines, BR15, and BR34) whose correlation with the skull metrics (red lines) were included in in ~30% of significantly different correlations in the two mutant mouse models. Scale bar = 1 mm.

Figure 6 shows the two brain linear distances and associated skull measures most frequently included in the brain-skull correlations that differed significantly between *Fgfr2*^+/*S252W*^ Apert syndrome mice and *Fgfr2c*^*C342Y*/+^ Crouzon syndrome mice. These brain metrics characterize the width of the posterior aspect of the brain including the cerebellum. The large majority of differences in brain skull integration between the two craniosynostosis models include associations between these brain measures and metrics that include all parts of the cranial vault and cranial base oriented along the superoinferior, mediolateral, and anteroposterior axes (Figure 6).

#### Unaffected littermates from two craniosynostosis mouse models show strongest differences in brain-skull integration

As a final assessment, we compared brain-skull integration patterns between unaffected littermates of the two craniosynostosis models. As noted in Materials and Methods, *Fgfr2*^+/*S252W*^ Apert syndrome mice are maintained on an inbred B6 background. *Fgfr2c*^*C342Y*/+^ Crouzon syndrome mice are maintained on a CD1 outbred background known to maximize heterogeneity (Chia et al., 2005). Of the 2,025 correlations among unique pairs of skull and brain metrics, 424 (20.94%) are significantly different between these groups. Of these differences, 108 indicate stronger correlation coefficients in the unaffected mice of the *Fgfr2*^+/+^ (B6) group while 316 of the differences are indicative of correlation coefficients that are of a greater magnitude in unaffected mice of the *Fgfr2c*^+/+^ (CD1) group. Since mice in the two groups used in this comparison do not carry an activated FGFR2 mutation associated with craniosynostosis, this comparison reveals differences in associations between brain and skull due to mouse strain/stock.

**Figure 7:**
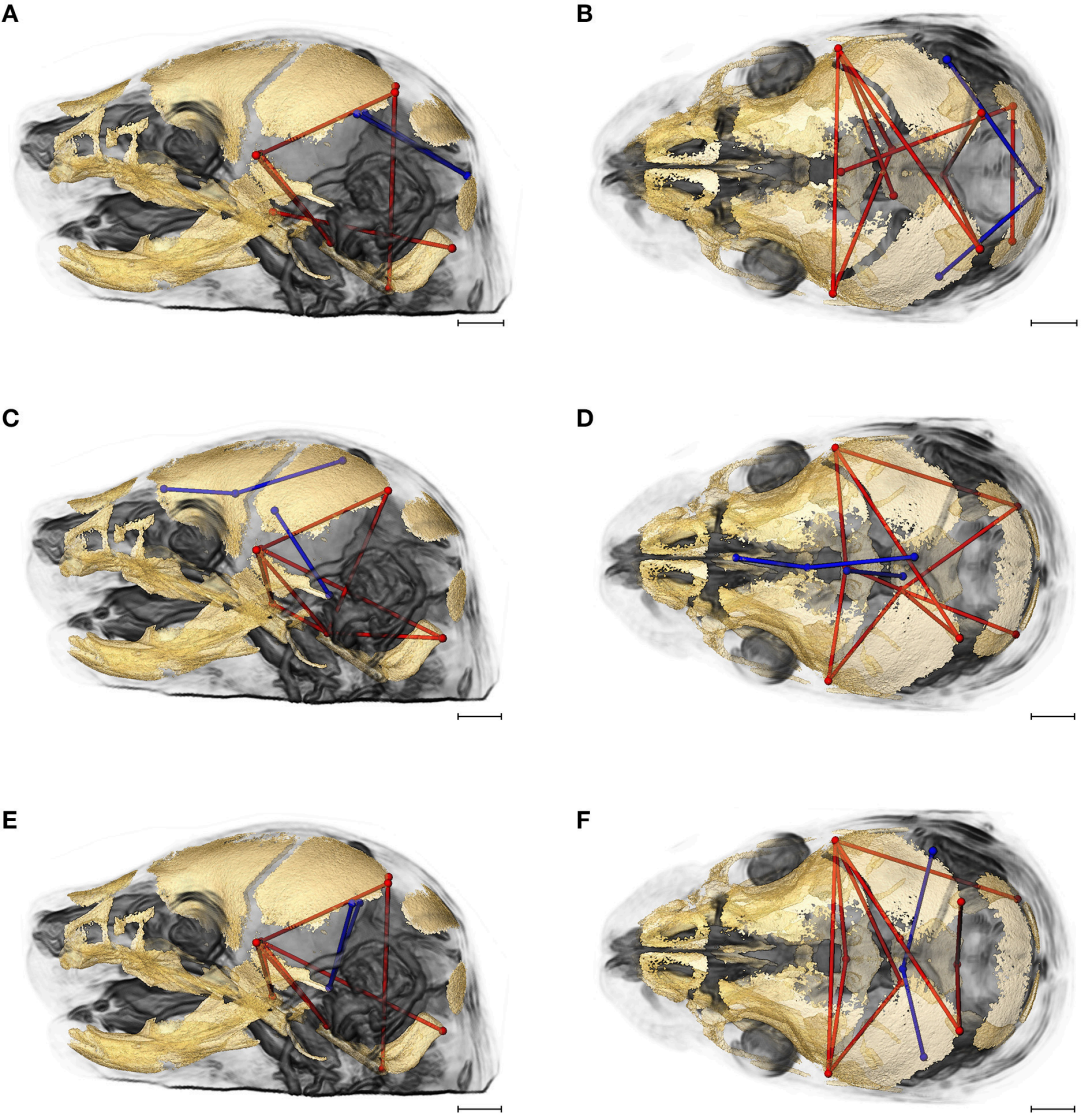
Brain (in blue) and skull (in red) linear distances whose associations were statistically significantly different between *Fgfr2c*^+/+^ and *Fgfr2*^+/+^ mice at E17.5 pictured on HRMRM and HRμCT reconstruction of a *Fgfr2c*^+/+^ mouse (**A**, lateral view; **B**, superoinferior view). **(A,B)** Two brain metrics [blue lines, BR15 (lpol&midcb) and BR34 (rpol&midcb) whose correlation with the skull metrics (red lines) were included in in ~14% of significantly different correlations in the two mutant mouse models]. **(C,D)** Three brain linear distances representing metrics associated with the corpus callosum BR39 (spcc&gcc), BR18 (obnp&gcc), BR1 (aptc&ac) are shown in blue. These brain metrics were involved in 10% of the total significant differences in correlations with specific skull measures (shown in red). **(E,F)** Two brain linear distances, BR30 (rpol&aptc) and BR8 (lpol&aptc) (in blue) were involved in an additional 5.60% of brain-skull correlations that were significantly different between CD1 and B6 mice. Skull linear distances are shown in red. The linesets represented in **(A–F)** represent nearly 30% of the correlations between brain and skull that were significantly different between CD1 and B6 mice. Scale bar = 1 mm.

Figures 7A,B provides summary illustrations of the measures most often involved in brain-skull correlations that were significantly different between the *Fgfr2*^+/+^ and the *Fgfr2c*^+/+^ (non-mutant) groups. Brain linear distances BR15 (lpol&midcb) and BR34 (rpol&midcb) were involved in 13.74% of the associations that showed significant differences (Figures 7A,B). These two brain linear distances were also those most commonly involved in significant differences in brain-skull integration between the two mutant models (Figure 6). Correlations between brain measures that summarize dimensions of the corpus collosum [BR39 (spcc&gcc), BR18 (obnp&gcc)] and the ventral cerebral surface (BR1 aptc&ac) and various measures of the vault (but only one cranial base measure) made up 10.43% of the associations that were significantly different between the two non-mutant groups (Figures 7C,D). Finally, the association of brain measures BR30 (rpol&aptc) and BR8 (lpol&aptc) that describe the height and width of the posterior aspect of the occipital lobe with anterior cranial vault measures were involved in an additional 5.6% of the significant differences in brain-skull integration for these groups (Figures 7E,F).

## Discussion

We used correlation matrices of linear distances estimated from the brain and skull of mouse models for craniosynostosis to statistically compare patterns of brain-skull integration among mice carrying mutations causative for craniosynostosis and their unaffected littermates. Our approach avoids problems inherent to superimposition methods used to estimate morphological integration and does not require the affine registration needed to combine brain measures from HRMRM images with skull measures from HRμCT images (see Methods of Analysis). Using relatively small samples we have shown how patterns of brain-skull integration vary among different groups, but determining why they vary (or persist unperturbed) will require alternate research strategies.

Many studies of skull integration test *a priori* hypotheses about localized differences in covariation among traits proposed to be important for the function, development or evolution of particular semiautonomous cranial trait complexes (Porto et al., 2009; Roseman et al., 2011), or more routinely, test the traditional hypothesis of increased covariation among traits that comprise the cranial vault, cranial base and facial skeleton. Martínez-Abadías et al. (2011) considered patterns of integration of cranial trait complexes based on developmental criteria by examining a hypothesis of covariance patterns that reflect the derivation of cells that contribute to cranial elements (either from neural crest or mesoderm) and on the basis of mode of ossification of cranial traits (either endochondral or intramembranous), but those efforts did not reveal significant findings. Studies of patterns of brain covariation have focused on the autonomy of brain connectivity networks to reveal mechanisms underlying their interactions or covariation of evolutionary morphology and the acquisition of particular cerebral functions or behaviors (Bruner, 2004; Balanoff et al., 2016).

Recognizing the developmental and structural correspondence between brain and skull (Richtsmeier et al., 2006; Nieman et al., 2012), we proposed a research design to evaluate covariation patterns of these two tissues during late prenatal development. We recognize that covariation patterns within the developing craniofacial complex most likely include additional and temporally varying tissue components and functioning spaces as development progresses. For example, proposed localized differences in covariation have been or could be analyzed to reveal developmental relationships between the facial prominences and brain (Parsons et al., 2011), the chondrocranium and forming cranial vault bones (Kawasaki and Richtsmeier, 2017a,b) and the nasal capsular cartilage, airway space, and midfacial bones (Martínez-Abadías et al., 2013a,b). As pointed out by Hallgrimsson et al. (2009), no matter what covariation patterns reveal at a particular time, these patterns may be replaced and erased as other key criteria for integration take their place during development. Identification of the mechanistic basis of any covariation patterns that signal hybrid-tissue units that function together or respond jointly to network-based regulatory signals will need to be verified by molecular work, and these mechanisms may be short-lived. Currently, knowledge of brain-skull covariation is observational (e.g., Richtsmeier et al., 2006; Bruner et al., 2015), but there are not enough data to test expectations grounded in theory, and a proper logic for defining the initiation of brain-skull covariation patterns and their change over time is sorely needed (Richtsmeier and Flaherty, 2013). Our analysis can help build hypotheses about the properties that structure covariation of brain and skull in typically developing individuals and those carrying mutations associated with structural birth defects.

Development of the brain is initiated with the differentiation of a specialized surface ectoderm, the neural plate that establishes the early brain by forming the neural tube. Skull development begins relatively later with the appearance of the chondrocranium (Kawasaki and Richtsmeier, 2017a) followed by proliferation and differentiation of osteoblast lineage cells that initiate intramembranous ossification of the frontal and parietal bones (Ishii, 2003; Long, 2011) and endochondral ossification of specific parts of the chondrocranium. As osteoblasts differentiate, cranial vault bones form on a scaffold established by the chondrocranium (Kawasaki and Richtsmeier, 2017a) and on the surface of the meninges, taking on the shape of the developing brain. It is unclear whether this is a passive positional correspondence, or if the shapes of the vault bones are informed (molecularly or biomechanically) by the brain surface through coordinated integration of signaling pathways (e.g., FGF, TGFb, WNT) via processes that are not currently understood (Richtsmeier and Flaherty, 2013; Neben and Merrill, 2015; Flaherty et al., 2016; Xavier et al., 2016; Lee et al., 2017). It is still unclear how genes individually or in concert enable and constrain the array of possible brain and skull morphologies during development. Answers to questions about how and when the developing brain and skull initiate interaction, the identity of the underlying mechanistic bases, and the evolutionary consequences of their integration, will help to sharpen our questions and to define hypotheses about their shared (and most likely shifting), integrated structure.

Results from our study indicate that brain-skull integration patterns are similar between each group carrying a mutation and their respective unaffected littermates, as very few linear distance pairs were shown to be significantly different. Of the 139 linear distances pairs that showed significant differences between *Fgfr2*^+/*S252W*^ Apert syndrome mice and their unaffected littermates and the 61 linear distance pairs that showed significant differences between *Fgfr2c*^*C342Y*/+^ Crouzon syndrome mice and their unaffected littermates, only 10 of these pairs were common to the two comparisons, suggesting that neither mutation causes profound differences in brain-skull integration and that the changes that do occur are mutation-specific, testifying to the uniqueness of each craniofacial syndrome. Though these FGFR2 mutations are responsible for profound changes in skull and brain morphology in human infants and in mouse models (Moosa and Wollnik, 2016), they fail to significantly disrupt the pattern of integration between brain and skull. If integration is a property of developing systems, our work advocates a brain-skull covariance structure driven by tight coordination between FGF/FGFR signaling and other pathways (i.e., BMP, MAPK, WNT, IHH, SHH) that are robust to these mutations. How dysmorphogenesis (sometimes severe) occurs but integration patterns remain, is fundamental to understanding how evolutionary processes control the range of possible anatomical phenotypes.

Direct comparison of the two mutant models, each on a different background, and direct comparison of the unaffected littermates in the two models reveal the largest number of statistically significant differences in morphological integration patterns, yet the magnitudes of brain-skull associations remain high in all groups. Even as genetic variation has potentially accrued in the CD1 stock, associations between brain and skull remain strong but move in novel directions, reflecting alternate developmental associations. The most likely explanation of these differences is genetic drift. Outbred stocks like CD1 are closed populations of genetically variable animals that are bred to maintain maximum heterozygosity (Rice and O'Brien, 1980; Chia et al., 2005). For any specific outbred colony, the degree of genetic heterogeneity depends on previous history and can range from almost zero to very extensive (Chia et al., 2005), while inbred stocks are homozygous at all loci. Relative to mutant and unaffected mice of the Apert sample, mutant and unaffected mice that comprise our Crouzon sample are larger in size (a trait common to outbred stocks; Chia et al., 2005), and quite different in brain and skull morphology [Figure 3 and additional data (not shown)]. More than half (*N* = 156) of the 277 linear distance pairs that were found to be significantly different in the comparison of *Fgfr2*^+/*S252W*^ Apert syndrome mice and *Fgfr2c*^*C342Y*/+^ Crouzon syndrome mice were also found to be significantly different in our comparison of the unaffected littermates of the two models. This suggests that nearly half the differences in brain-skull integration between *Fgfr2*^+/*S252W*^ Apert syndrome mice and *Fgfr2c*^*C342Y*/+^ Crouzon syndrome mice are due to the developmental architecture and variation in the “background” genomes of these particular samples, while the remaining differences are due to the interaction of each craniosynostosis-associated mutation with the respective genomes of those strains.

Examples of how the comparative study of mouse strains has revealed that genetic changes underlie phenotypic changes are many and diverse, and include variation in phenotypic response to single mutations on varying genetic backgrounds (Twigg et al., 2009), differences in postcranial cortical bone structure response to biomechanical input in different mouse strains (Wallace, 2013; Wallace et al., 2015), and the non-equivalence of additive genetic effects underlying defining phenotypic characteristics of specific strains (Percival et al., 2016), to name a few. These studies provide evidence of varying developmental mechanisms that can generate or constrain variation, quantified as differences in the ways that specific genotypes respond developmentally to environmental inputs by building phenotypes. Our findings suggest that certain differences in craniofacial development across mouse strains can be produced either by a change in the intensity of covariation patterns, or changes in the actual patterns of covariation with little change in the strength of the relationships.

Study of disease states, or “nature's experiments” (Pruzansky, 1982), inform us about the complexities of normal development. Investigation of the processes underlying brain-skull integration in FGFR-related craniosynostosis conditions will involve investigation of: the interaction of FGF/FGFR signaling with other major signaling pathways (i.e., BMP, MAPK, WNT, IHH, SHH) that influence brain and skull formation; the biomechanical interactions of brain and skull growth (Moss and Young, 1960; Garzón-Alvarado et al., 2013; Lee et al., 2015, 2017); and the relationship between primary shape changes associated with the direct effects of FGFR mutations and secondary shape changes triggered by the indirect effects of changes in covariation between the brain and skull (Martínez-Abadías et al., 2011). Relevant gene families and the signaling systems in which they operate likely evolved together along with the morphological, structural and functional variation that they foster (Richtsmeier and Flaherty, 2013). It is possible that those networks oversee the conformity of brain and skull shape by cooperatively managing their development.

Brain-skull integration is a fundamental property that likely contributed to the evolution of vertebrate head phenotypes. The mechanistic basis for observed patterns of covariation explains the coordination of these tissues in evolution, development, and disease. The challenge is to transcend traditional anatomic-based classifications in development and to identify autonomous units (most likely mixed-tissue) that cooperate structurally, function together, or exhibit synchronized responses to regulatory networks. Only then can we begin to understand covariation generating mechanisms in development.

## Author contributions

JR conceived the project and designed experiments with EJ. TS and TR designed CT imaging approaches and protocols. TS designed MRM imaging approaches and protocols. SM performed and analyzed MRM experiments under the supervision of TN, reconstructed CT and MRM images, collected CT and MRM data, and created all illustrations. SM and JR designed statistical analyses, analyzed data, and interpreted results. SM, TS, TN, EJ, TR, and JR contributed to writing the manuscript.

### Conflict of interest statement

The authors declare that the research was conducted in the absence of any commercial or financial relationships that could be construed as a potential conflict of interest.

## Supplementary material

Supplementary Figure 1 Results of PCA analyses of shape based on linear distances estimated among landmarks for skull and brain. **(A,B)** Scatter plots of individual scores based on PCA of skull shape. **(A)** Distribution of *Fgfr2c*^*C342Y*/+^ mutant mice and unaffected littermates (*Fgfr2c*^+/+^) along first and second Principal Components axes (PC1 and PC2) for skull shape. **(B)** Distribution of *Fgfr2*^+/*S*252*W*^ Apert syndrome mice and unaffected littermates (*Fgfr2c*^+/+^) along first and second Principal Components axes (PC1 and PC2) for skull shape. **(C,D)** Scatter plots of individual scores based on PCA of brain shape. **(C)** Distribution of *Fgfr2c*^*C342Y*/+^ mutant mice and unaffected littermates (*Fgfr2c*^+/+^) along first and second Principal Components axes (PC1 and PC2) for brain shape. **(D)** Distribution of *Fgfr2*^+/*S252W*^ Apert syndrome mice and unaffected littermates (*Fgfr*^+/+^) along first and second Principal Components axes (PC1 and PC2) for brain shape.

Supplementary Table 1 Correlation matrices across all samples. Linear distances are labeled as representing a metric between two brain landmarks (BR#) or between two landmarks on bones of the skull (BO#). Codes for the landmarks that serve as endpoints for these linear distances are found in Supplementary Table 2.

Supplementary Table 2 Listing of all brain (BR#) and skull (BO#) metrics used in analysis and their definition as determined by landmarks that define the linear distance endpoints.
