# Supplementary figures and images for: Integration of Brain and Skull in Prenatal Mouse Models of Apert and Crouzon Syndromes
Source: Front Hum Neurosci. 2017 Jul 25;11:369. doi: 10.3389/fnhum.2017.00369 (PMC5525342; doi:10.3389/fnhum.2017.00369)

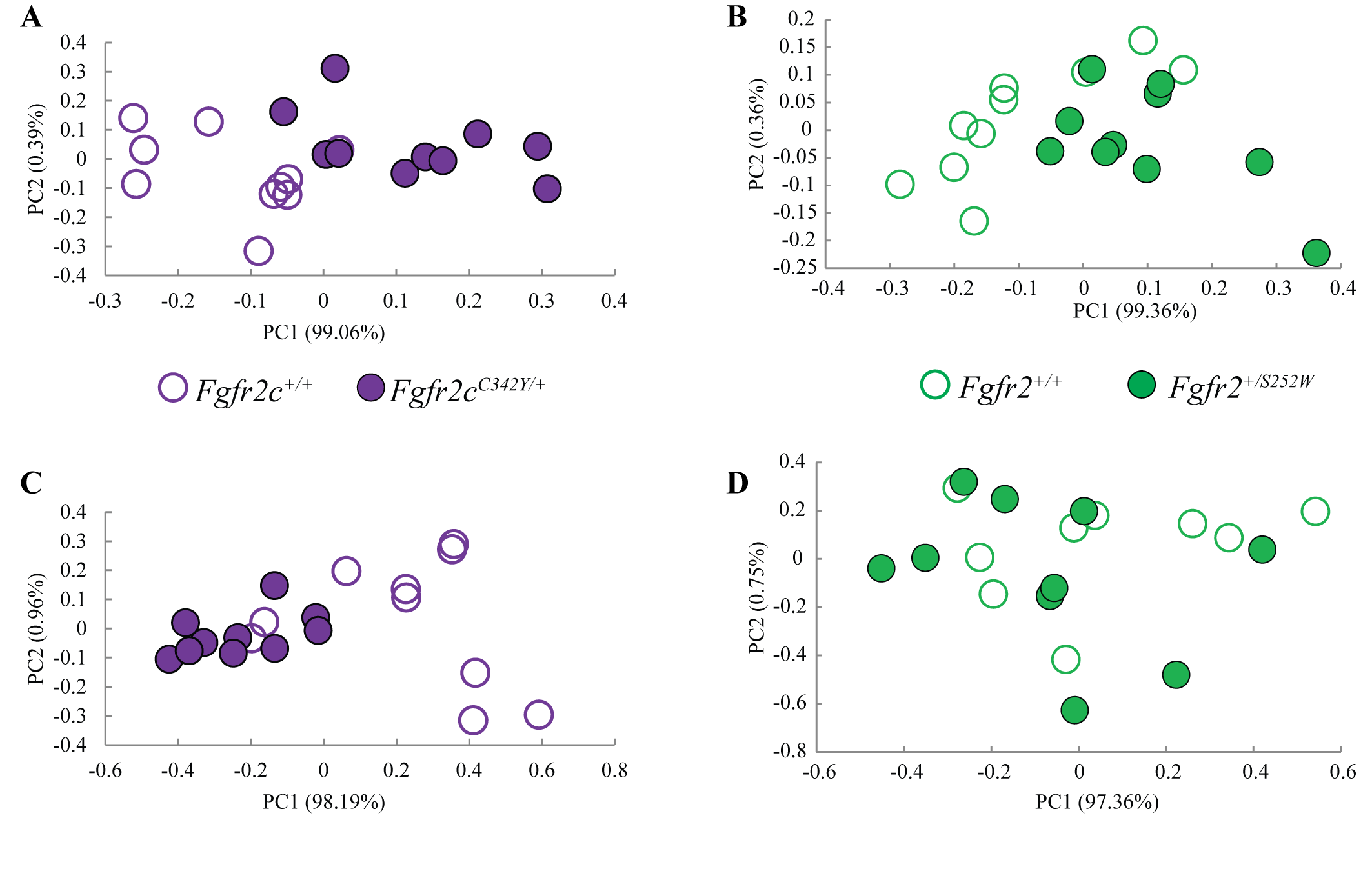

Supplement: Supplementary Figure 1 — Results of PCA analyses of shape based on linear distances estimated among landmarks for skull and brain. (A,B) Scatter plots of individual scores based on PCA of skull shape. (A) Distribution of Fgfr2cC342Y/+ mutant mice and unaffected littermates (Fgfr2c+/+) along first and second Principal Components axes (PC1 and PC2) for skull shape. (B) Distribution of Fgfr2+/S252W Apert syndrome mice and unaffected littermates (Fgfr2c+/+) along first and second Principal Components axes (PC1 and PC2) for skull shape. (C,D) Scatter plots of individual scores based on PCA of brain shape. (C) Distribution of Fgfr2cC342Y/+ mutant mice and unaffected littermates (Fgfr2c+/+) along first and second Principal Components axes (PC1 and PC2) for brain shape. (D) Distribution of Fgfr2+/S252W Apert syndrome mice and unaffected littermates (Fgfr+/+) along first and second Principal Components axes (PC1 and PC2) for brain shape. [file Image1.tif]
