# Supplementary material for: Integration of Brain and Skull in Prenatal Mouse Models of Apert and Crouzon Syndromes
Source: Front Hum Neurosci. 2017 Jul 25;11:369. doi: 10.3389/fnhum.2017.00369 (PMC5525342; doi:10.3389/fnhum.2017.00369)
Supplement: Supplementary Table 2 — Listing of all brain (BR#) and skull (BO#) metrics used in analysis and their definition as determined by landmarks that define the linear distance endpoints. [file Table2.DOCX]

| Skull linear distance | skull landmarks that serve as endpoints of linear distance | brain linear distance | brain landmarks that serve as endpoints of linear distance |
| --- | --- | --- | --- |
|  |  |  |  |
| BO1 | bas&amsph | **BR1** | aptc&ac |
| BO2 | loci&amsph | **BR2** | gcc&ac |
| BO3 | loci&bas | **BR3** | gcc&aptc |
| BO4 | lpto&amsph | **BR4** | lobc&ac |
| BO5 | lpto&bas | **BR5** | lobc&aptc |
| BO6 | lpto&loci | **BR6** | lobc&gcc |
| BO7 | lsqu&amsph | **BR7** | lpol&ac |
| BO8 | lsqu&bas | **BR8** | lpol&aptc |
| BO9 | lsqu&loci | **BR9** | lpol&gcc |
| BO10 | lsqu&lpto | **BR10** | lpol&lobc |
| BO11 | lsyn&amsph | **BR11** | midcb&ac |
| BO12 | lsyn&bas | **BR12** | midcb&aptc |
| BO13 | lsyn&loci | **BR13** | midcb&gcc |
| BO14 | lsyn&lpto | **BR14** | midcb&lobc |
| BO15 | lsyn&lsqu | **BR15** | midcb&lpol |
| BO16 | roci&amsph | **BR16** | obnp&ac |
| BO17 | roci&bas | **BR17** | obnp&aptc |
| BO18 | roci&loci | **BR18** | obnp&gcc |
| BO19 | roci&lpto | **BR19** | obnp&lobc |
| BO20 | roci&lsqu | **BR20** | obnp&lpol |
| BO21 | roci&lsyn | **BR21** | obnp&midcb |
| BO22 | rpto&amsph | **BR22** | robc&ac |
| BO23 | rpto&bas | **BR23** | robc&aptc |
| BO24 | rpto&loci | **BR24** | robc&gcc |
| BO25 | rpto&lpto | **BR25** | robc&lobc |
| BO26 | rpto&lsqu | **BR26** | robc&lpol |
| BO27 | rpto&lsyn | **BR27** | robc&midcb |
| BO28 | rpto&roci | **BR28** | robc&obnp |
| BO29 | rsqu&amsph | **BR29** | rpol&ac |
| BO30 | rsqu&bas | **BR30** | rpol&aptc |
| BO31 | rsqu&loci | **BR31** | rpol&gcc |
| BO32 | rsqu&lpto | **BR32** | rpol&lobc |
| BO33 | rsqu&lsqu | **BR33** | rpol&lpol |
| BO34 | rsqu&lsyn | **BR34** | rpol&midcb |
| BO35 | rsqu&roci | **BR35** | rpol&obnp |
| BO36 | rsqu&rpto | **BR36** | rpol&robc |
| BO37 | rsyn&amsph | **BR37** | spcc&ac |
| BO38 | rsyn&bas | **BR38** | spcc&aptc |
| BO39 | rsyn&loci | **BR39** | spcc&gcc |
| BO40 | rsyn&lpto | **BR40** | spcc&lobc |
| BO41 | rsyn&lsqu | **BR41** | spcc&lpol |
| BO42 | rsyn&lsyn | **BR42** | spcc&midcb |
| BO43 | rsyn&roci | **BR43** | spcc&obnp |
| BO44 | rsyn&rpto | **BR44** | spcc&robc |
| BO45 | rsyn&rsqu | **BR45** | spcc&rpol |
